# P-element Somatic Inhibitor Protein Binding a Target Sequence in *dsx* Pre-mRNA Conserved in *Bombyx mori* and *Spodoptera litura*

**DOI:** 10.3390/ijms20092361

**Published:** 2019-05-13

**Authors:** Yao Wang, Qin Zhao, Qiu-Xing Wan, Kai-Xuan Wang, Xing-Fu Zha

**Affiliations:** 1 State Key Laboratory of Silkworm Genome Biology, Biological Science Research Center, Southwest University, Beibei, Chongqing 400715, China; tifa1224@163.com (Y.W.); 15516767013@163.com (Q.Z.); Wanqiux@outlook.com (Q.-X.W.); wkx7418@email.swu.edu.cn (K.-X.W.); 2 Chongqing Key Laboratory of Sericultural Science, Southwest University, Chongqing 400715, China; 3 Chongqing Engineering and Technology Research Center for Novel Silk Materials, Southwest University, Chongqing 400715, China

**Keywords:** *Bombyx mori*, *Spodoptera litura*, sex determination, BmPSI, KH_1 motif, electrophoretic mobility shift assay (EMSA), isothermal titration calorimetry (ITC), circular dichroism (CD) spectroscopy

## Abstract

*Bombyx mori doublesex* (*Bmdsx*) functions as a double-switch gene in the final step of the sex-determination cascade in the silkworm *Bombyx mori*. The P-element somatic inhibitor (PSI) protein in *B. mori* interacts with *Bmdsx* pre-mRNA in CE1 as an exonic splicing silencer to promote male-specific splicing of *Bmdsx*. However, the character of the interaction between BmPSI and *Bmdsx* pre-mRNA remains unclear. Electrophoretic mobility shift assay (EMSA) results showed that the four KH_1 motifs in BmPSI are all essential for the binding, especially the former two KH_1 motifs. Three active sites (I116, L127, and IGGI) in the KH_1 motif were found to be necessary for the binding through EMSA, circular dichroism (CD) spectroscopy, and isothermal titration calorimetry (ITC). The PSI homologous protein in *S. litura* (SlPSI) was purified and the binding of SlPSI and CE1 was verified. Compared with BmPSI, the mutant SlPSI proteins of I116 and IGGI lost their ability to bind to CE1. In conclusion, the binding of PSI and *dsx* pre-mRNA are generally conserved in both *B. mori* and *S. litura*. These findings provide clues for sex determination in Lepidoptera.

## 1. Introduction

Sex determination is a fundamental process but has been found to vary among different taxa. Sex determination mechanisms in the silkworm *Bombyx mori* differ from those in *Drosophila melanogaster*, especially the upstream of *dsx* which functions as a double-switch gene in the final step of the sex-determination cascade. In 2001, a double-switch gene, *Bmdsx*, was found at the final step in the sex-determination cascade of *B. mori* [1,2]. When binding to a particular sequence splicing repressor, the female-specific splice sites in *Bmdsx* pre-mRNA are inhibited, leading to the male-specific alternative splicing of *Bmdsx* [3,4]. A series of mutation analyses using an in vivo splicing assay system identified three distinct sequences (CE1, CE2, and CE3) in exon 4 to be exonic splicing silencers responsible for male-specific splicing of *Bmdsx*. The CE1 sequence was shown to bind to the nuclear protein BmPSI. Using UAA repeats to replace CE1 inhibited the binding of BmPSI to the RNA and caused female-specific splicing of *Bmdsx* in male cells [5]. The transgenic knockout strain of *BmPSI* had a defective gonad in males [6]. Then, the interaction between BmPSI and CE1 was shown to be essential for sex determination of *B. mori* by silencing the female-specific alternative splicing of *Bmdsx*. Hence, research on binding characteristics will contribute to the discovery of the mechanisms underlying these alternative splicing processes during sex determination in many species.

BmPSI has four KH_1 motifs. The hnRNP K-homology (KH) motif has been shown to participate in sequence-specific recognition of target RNA in many species [7,8]. For example, the human PSI homolog KSRP contains four KH domains and participates in the alternative splicing of neuron-specific *c-src* pre-mRNA [9,10]. Furthermore, *Drosophila* PSI regulates the splicing of *P-element transposase* pre-mRNA by binding a pseudo-splice site upstream of the authentic splice site using four KH motifs [11,12,13]. Though there have been a few previous studies regarding these special proteins with multiple KH motifs, there is still little known about the detail contribution of the KH_1 motif and the key amino acids, except for the Ile-Gly-X2-Gly-X2-Ile structure, which is conservative in the KH_1 domain [14,15]. BmPSI contains four KH_1 motifs for binding RNA and there is a certain interaction between BmPSI and CE1. Thus, it is a good model protein in which to explore the contribution of the KH motif to recognize and bind RNA.

## 2. Results

### 2.1. Four KH_1 Motifs Play Important Roles in the Interaction Between BmPSI and CE1, Especially the KH_1-1 Motif and KH_1-2 Motif

**Figure 1:**
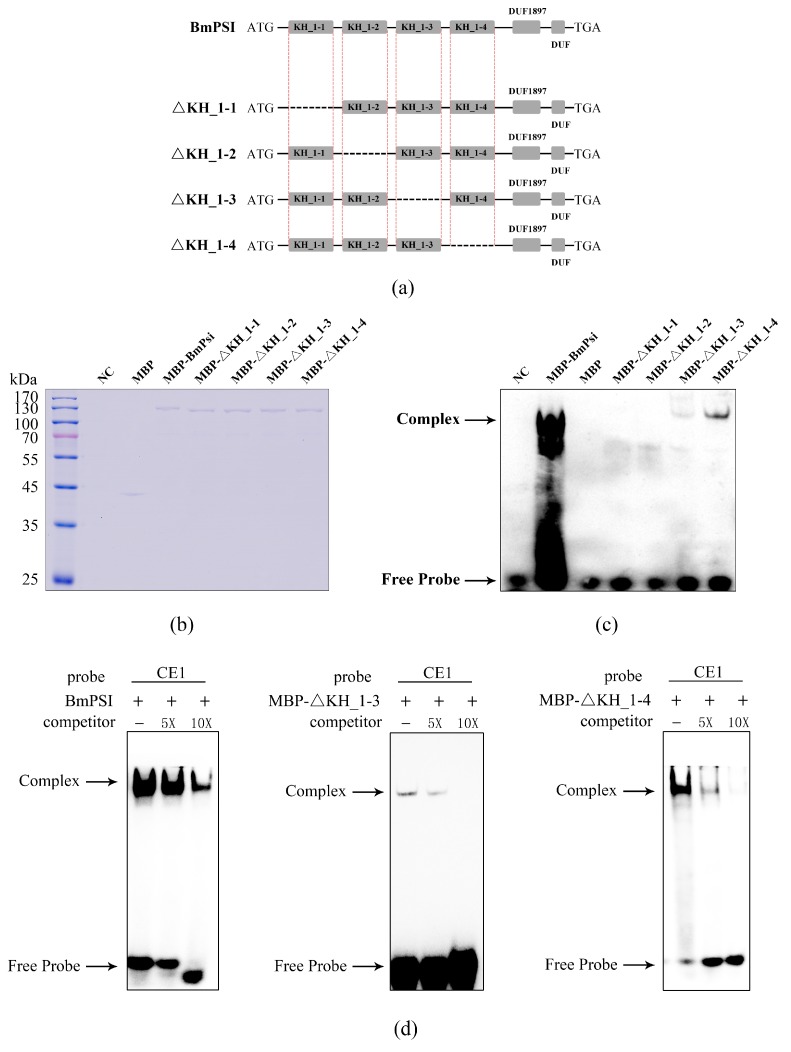
Structure and binding ability of BmPSI and truncation proteins: (**a**) Comparison among BmPSI and four truncation proteins used for the binding experiment. “DUF” means “domain of unknown function”. (**b**) The dosage of these proteins. “NC” means “negative control”. (**c**) Binding ability of these proteins with CE1. Buffer and maltose binding protein (MBP) were set as the negative control and wild-type BmPSI was used as the positive control. (**d**) Cold competition electrophoretic mobility shift assay (EMSA) experiment between CE1 and BmPSI, MBP-ΔKH_1-3, and MBP-ΔKH_1-4 proteins.

According to the sequence of *BmPSI*, the tertiary structure of BmPSI was predicted to contain four KH_1 motifs and two other motifs of unknown function. The KH_1 motif was found to be an RNA binding motif in vitro. Therefore, we speculated that these four KH_1 motifs play important roles in the interaction between BmPSI and CE1. We purified maltose binding protein (MBP), wild-type BmPSI, and four truncate BmPSI proteins that respectively truncated KH_1-1, KH_1-2, KH_1-3, and KH_1-4 (Figure 1a). The concentrations of these proteins were adjusted to be identical to that of a bicinchoninic acid assay (BCA), and final concentrations of the adjustment were confirmed by sodium dodecyl sulfate-polyacrylamide gel electrophoresis (SDS-PAGE) (Figure 1b). After the concentrations of these six proteins were adjusted uniformly, they were used for an electrophoretic mobility shift assay (EMSA) (Figure 1c). As shown by the EMSA, MBP cannot bind to CE1, and the four mutant proteins almost lost their ability to interact with CE1, especially the ΔKH_1-1 and ΔKH_1-2, compared with the wild-type BmPSI, which was used as a positive control. In order to eliminate the effect of nonspecific binding, wild-type BmPSI, MBP- ΔKH_1-3, and MBP- ΔKH_1-4 proteins were used in a cold competition EMSA experiment (Figure 1d). When the concentration of the competitive probe was aggrandized, the signal intensity of the complex decreased sharply. These results showed that the interaction between CE1 and these three proteins are specific. Hence, we deduced that these four KH_1 motifs participate in the interaction between BmPSI and CE1. In addition, there was absolutely no binding signal found in the lines of MBP- ΔKH_1-1 and MBP- ΔKH_1-2, which was in contrast to the stable binding signal in the lines of MBP- ΔKH_1-3 and MBP- ΔKH_1-4. This result indicates that the KH_1-1 and KH_1-2 motifs play more important roles than the other two KH_1 motifs in the binding affinity of BmPSI.

### 2.2. Important Amino Acids in the KH_1-1 Motif of BmPSI Found in the Interaction between BmPSI and CE1

**Figure 2:**
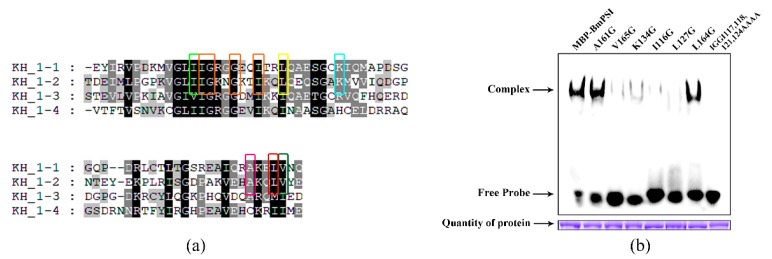
Important amino acids in the KH_1-1 motif of BmPSI: (**a**) Comparison among amino acid sequences of the KH_1 motifs in BmPSI. The black color represents that amino acids are completely identical. The gray color represents that partial amino acids are identical. Different colored boxes represent different potential key amino acid sites for the binding. (**b**) After the concentrations were adjusted uniformly, EMSA was performed on wild-type BmPSI and seven amino acid mutants of BmPSI.

We have discussed how KH_1-1 and KH_1-2 play important roles in the interaction between BmPSI and CE1. Through the comparison of the four KH_1 motifs of BmPSI, seven potential key amino acids were predicted (Figure 2a). After cloning these amino acid mutant BmPSI proteins, the proteins were purified with the wild-type BmPSI for EMSA (Figure 2b). EMSA results indicated that five types of mutant proteins lost most of their binding ability compared with the wild-type BmPSI, especially I116G, L127G, and the mut IGGI.

**Figure 3:**
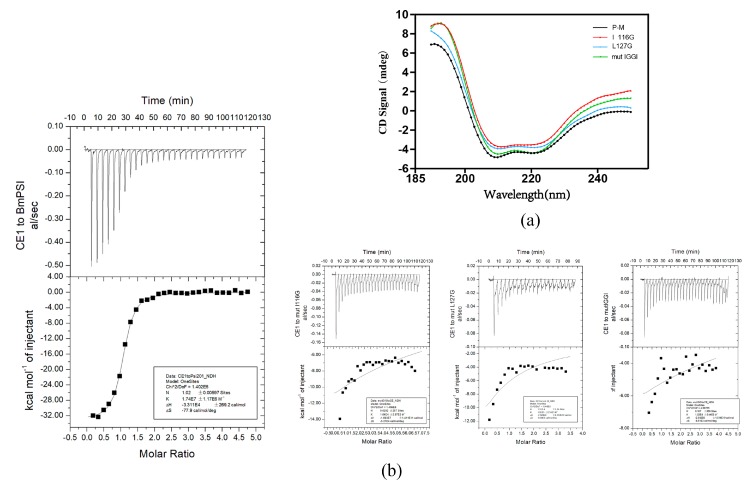
Two amino acids in the KH_1-1 motif of BmPSI play important roles in the binding with CE1: (**a**) The secondary structures of wild-type BmPSI and three kind of amino acid mutant proteins detected with circular dichroism (CD) spectroscopy. (**b**) The RNA binding affinity of three kinds of amino acid mutant proteins and wild-type BmPSI were assessed, showing different exports of isothermal titration calorimetry (ITC) under the same conditions.

To determine whether these three amino acid mutant proteins transform the secondary structure of BmPSI, circular dichroism (CD) spectroscopy was performed on the wild-type BmPSI and these three mutant proteins (Figure 3a). The results suggest that the three mutant proteins and the wild-type BmPSI have similar patterns. There are two minima at 208 and 222 nm in the result, which is considered to be the norm for α-helices [16]. This suggests that these amino acid mutations did not influence the interaction process by altering the secondary structure of BmPSI. To verify that these three amino acid mutants did lose their ability to bind CE1, isothermal titration calorimetry (ITC) was performed (Figure 3b) [17,18]. Firstly, the wild-type BmPSI was analyzed to explore the appropriate conditions for the experiment. Then, the detailed energetic profile of the interaction between BmPSI and CE1 was obtained (Table 1). The average K value of 1.74 × 10^7^ (*n* =1.02) of the CE1/BmPSI system indicates that there is a strong interaction between BmPSI and CE1.

The three mutant proteins were analyzed individually with the same conditions grouped from the experiment of BmPSI and CE1. From the ITC results, we found that the ITC data of these three amino acid mutant proteins could not fit the model properly, indicating that these mutant proteins did lose their ability to interact with CE1.

### 2.3. The Combination of PSI and CE1 is Conserved in Spodoptera Litura

**Figure 4:**
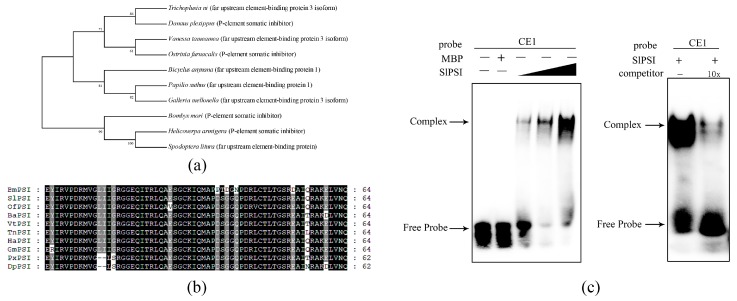
The combination of PSI and CE1 is conserved in *Spodoptera litura*: (**a**) A phylogenic tree based on the PSI of 10 species. (**b**) Conservation analysis of KH_1 motifs in these 10 species. The black color represents that amino acids are completely identical. The gray color represents that partial amino acids are identical. (**c**) SlPSI concentration gradient EMSA and cold competition EMSA.

Among the three kinds of amino acid mutants, the two which were not IGGI were found close to the Ile-Gly-X2-Gly-X2-Ile structure, which was conserved in the KH_1 motifs. Hence, we considered that the two amino acids near the Ile-Gly-X2-Gly-X2-Ile structure strongly influenced the combination of PSI and RNA in not only *B. mori* but also other species. A phylogenic tree was performed with these sequences of homolog PSI in ten species (Figure 4a). It was shown that BmPSI, SlPSI, and *Helicoverpa armigera* PSI (HaPSI) were clustered into the same clade. From the conservative analysis of KH_1 motifs in these ten species, it was found that the KH_1-1 motif of SlPSI was similar to the KH_1-1 of BmPSI (Figure 4b). SlPSI was selected as the experimental subject to verify the importance of key amino acids for the combination of PSI and CE1. It was confirmed that the CE1 exists conservatively in the female-specific splicing of *Sldsx* in *S. litura*. Full length coding sequence (CDS) of *SlPSI* was cloned and the SlPSI was purified with Ni-NTA and gel filtration chromatography. Cold competition EMSA and SlPSI concentration gradient EMSA were used to show that the combination of PSI and CE1 was also conserved in *S. litura* (Figure 4c).

### 2.4. Identifying a Key Amino Acid for the Combination of SlPSI and CE1

**Figure 5:**
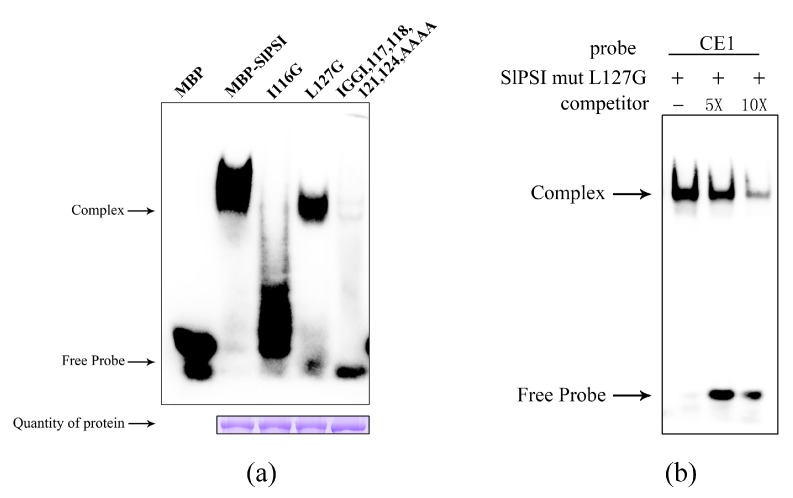
EMSA of wild-type SlPSI and three kinds of potentially key amino acid mutant SlPSI proteins. (**a**) MBP-BmPSI was set as the positive control. The signal intensity of the complex stood for the abilities of different proteins to interact with the CE1 probe. (**b**) Cold competition EMSA experiment between CE1 and SlPSI mutL127G.

Three types of amino acid mutations (L116I, L127G, and IGGI) were added into SlPSI. The mutant proteins were purified and the concentrations of mutant proteins were adjusted consistently for EMSA (Figure 5a). The results showed that mut IGGI can weakly bind to CE1, and that I116G lost most of its ability to interact with CE1 compared to SlPSI. The L127G also lost a lot of its binding capacity but this was not as conspicuous as with I116G. In order to eliminate the effect of nonspecific binding, the SlPSI mutL127G protein was used in a cold competition EMSA experiment (Figure 5b). When the concentration of the competitive probe was aggrandized, the signal intensity of the complex decreased sharply. This result showed that the interaction between CE1 and SlPSI mutL127G is specific. To summarize, we found two key amino acids in the KH-1-1 motif of SlPSI. One was the conservative key structure Ile-Gly-X2-Gly-X2-Ile of the KH_1 motif that has been reported previously [15] and the other was the amino acid I116, which is close to Ile-Gly-X2-Gly-X2-Ile. This is a new discovery of the key amino acid in proteins containing the KH_1 motif for binding its target RNA.

## 3. Discussion

BmPSI contains four KH_1 motifs and it is considered to be an informative model for exploring how these KH motifs contribute to RNA binding and recognition in these proteins with multiple KH motifs. The EMSA of BmPSI and four truncate BmPSI proteins showed that the four KH_1 motifs participate in the interaction between BmPSI and CE1, in which the KH_1-1 and KH_1-2 play more important roles in the combination process. Through sequence alignment, EMSA, CD spectroscopy, and ITC, we found three potentially key amino acids in the KH_1-1 motif of BmPSI. After verifying the interaction of SlPSI and CE1, these three kinds of amino acid mutations were cloned into SlPSI. Except for the L127G mutation, we found that the I116G and IGGI mutations lost most of their binding ability compared with the wild-type SlPSI.

*Drosophila* PSI requires multiple tandem KH domains for specific and high-affinity recognition of substrate RNA [11]; however, the KH_1-1 and KH_1-2 motifs of BmPSI play more important roles in the interaction of BmPSI and *Bmdsx* pre-mRNA compared with the other two KH_1 motifs. Differences in the binding ability of the L127 key site in BmPSI and SlPSI suggest that there are a lot of differences between these two relatively close lepidopterans. The binding affinity of mutant I116G sharply decreased in BmPSI and SlPSI, indicating that this is a key site in the KH_1 motif for the binding process. Furthermore, I116G is stably conserved in Lepidoptera, which indicates it is a key amino acid for binding affinity in proteins containing KH_1 motifs, just as EMSA confirmed it was. I116 is isoleucine, which is tightly close to the Ile-Gly-X2-Gly-X2-Ile structure. These discoveries provide further insight into how KH_1 motifs contribute to the combining of a protein and its target RNA sequence.

The isoforms of sex-specific alternative splicing of *Bmdsx* are translated into sex-specific BmDSX proteins which lead to differences in the expression of sex-specific genes, and, thus, sexual differentiation [19,20]. BmPSI is the most important part of the exonic splicing silencer complex for binding *Bmdsx* pre-mRNA with CE1 to promote male-specific splicing of *Bmdsx* [21,22]. This is the only mechanism known as being closely upstream of *Bmdsx* in the sex determination cascade of *B. mori.* The researchers could not generate a gender reversal strain through genetic modification of *Bmdsx* or the knockout of *Bmpsi*. Two possible reasons for this are that *Bmdsx* leads the expressions of many sex-specific genes and *Bmpsi* participates in not only sex determination but also other key pathways in *B. mori*. Therefore, a slight change in *Bmdsx* or *Bmpsi* would cause disorder in transgene stains. Thus, the key amino acid sites of the interaction between BmPSI and CE1 could be used to establish transgenic strains, in which the transgenic BmPSI loses its capacity to bind to CE1 but retains its other functions.

From an evolutionary perspective, the four conserved KH_1 motifs in SlPSI and CE1 in female specific splicing of *Sldsx* provide strong evidence of conserved structures for essential components of the sex determination process in Lepidoptera [23]. Furthermore, the conserved interaction of SlPSI and CE1 indicates functional conservation of essential components in the sex determination process in Lepidoptera. In addition, the discovery of this key site for the binding in both BmPSI and SlPSI not only contributes a lot to future research on sex determination of lepidopterans but also provides a theoretical basis for gender reversal in *Bombyx mori* [24,25].

## 4. Materials and Methods

### 4.1. Construction of Recombinant Expression Vectors

The pET His_6_ MBP TEV LIC cloning vector is an LIC N-terminal fusion vector for *E. coli* expression which contains a maltose binding protein. The wild type *Bmpsi* sequence was cloned into this vector behind a (His)_6_ affinity tag by LIC cloning. Four different KH_1 region truncations of *Bmpsi* were made with overlapping PCR. Seven point mutations in the KH_1-1 motif of *Bmpsi* were added with site-directed mutagenesis into the recombinant vector containing *Bmpsi* (Table 2).

The sequence of *Slpsi* was downloaded from NCBI and was predicted to be the *S. litura* far upstream element-binding protein (LOC 111350908). According to the sequence information on NCBI, we designed a pair of primers to clone the fragment of *Slpsi* into the pET His_6_ MBP TEV LIC cloning vector. Three kinds of point mutation in the KH_1-1 motif of *Slpsi* were added with site-directed mutagenesis into the recombinant vector (Table 3).

### 4.2. Preparation of Single Stranded RNA

Two kinds of CE1 single-stranded RNA were prepared for EMSA and ITC. One kind of CE1 RNA was labeled with Biotin on its 5′ end and 3′ end. The sequence of the single-stranded RNA was the same as the sequence of CE1 in the female-specific splicing of *Bmdsx* (5′-uuaauaauauaaguggugua-3′). The RNA probe was compounded by the Beijing Genomics Institute and purified with ion exchange HPLC. The RNA was dissolved in a buffer (20 mM Tris, 20 mM NaCl), which was used to purify the different kinds of protein.

### 4.3. The Overexpression and Purification of Different Kinds of Proteins

Transetta (DE3) Chemically Competent Cell was used to overexpress the wild-type BmPSI, the wild-type SlPSI, the four KH_1 region truncation proteins of BmPSI, the seven amino acid mutants of BmPSI proteins, and the three amino acid mutants of SlPSI proteins with the recombinant vectors discussed above. We overexpressed the maltose-binding protein with the original expression vector as a negative control. The purification of every kind of protein needed 2 L cells in LB media at 37 °C with an absorbance at 600 nm of 0.5. Then, we added Isopropyl β-D-Thiogalactoside (IPTG)to a final concentration of 1 mM and cultured the cells at 37 °C for another 4 h. After cell culturing, the cells were gathered and crushed with multi-gelation three times and ultra-sonicated (40%, 30 min). The supernatant was separated using centrifugation (15,000× *g*, 10 min). A series of buffers with different concentrations of imidazole were used to wash the HiTrap Chelating where the supernatant was loaded. The buffer containing the target protein was passed through gel filtration chromatography, which made it purer. Finally, we retrieved the pure target protein with the (His)_6_ -MBP-TEV component.

### 4.4. Bioinformatics Analysis

The sequences of the four KH_1 motifs in BmPSI were compared to find out the potential key amino acids which are conservative in KH_1-1and KH_1-2 but not KH_1-3 and KH_1-4 [26,27,28]. The PSI sequences of ten species were downloaded from NCBI to construct a phylogenic tree [29,30] and the sequences of the four KH_1 motifs from BmPSI were compared by multiple sequence alignment.

### 4.5. Electrophoretic Mobility Shift Assay

Electrophoretic mobility shift assay can be used to determine binding affinity, specificity, and stoichiometry of the RNA/protein interaction [31,32,33]. In this article, we used EMSA experiments in different ways which were designed to accommodate two different experimental objectives. A normal EMSA was used to verify the interaction between SlPSI and CE1 with cold competition EMSA and SlPSI concentration gradient EMSA. The other kind of EMSA was designed to compare the binding affinity with RNA of different proteins under the same conditions. At first, the concentrations of proteins in every group were adjusted consistently with BCA assay and SDS-PAGE. Then, the proteins were experimented on under exactly the same conditions. As a result, the signal intensity of the complex stood for the abilities of different proteins to interact with the RNA probe. One group of proteins included MBP, MBP-BmPSI, and four KH_1 region truncate BmPSI proteins, which were set to compare the binding abilities of different KH_1 motifs. Another group of proteins included MBP-BmPSI and the seven amino acid mutant BmPSI proteins, which were set to show the importance these key amino acids had in the interaction between BmPSI and CE1. The third group of proteins included MBP-SlPSI and the three amino acid mutant SlPSI proteins, which were set to detect the conservative property of the key amino acids we found in BmPSI for the combination of PSI and CE1 in other species.

### 4.6. CD Wavelength Scans

Circular dichroism spectroscopy is a technique which is widely used to detect secondary structure composition. The wild-type BmPSI protein and the three kinds of amino acid mutations of BmPSI, which were important to the interaction between BmPSI and CE1, were experimented on for these secondary structures with CD wavelength scans. Before the CD wavelength test, the concentrations of different proteins were adjusted consistently with a BCA protein assay kit and SDS-PAGE.

### 4.7. Isothermal Titration Calorimetry

Isothermal titration calorimetry is always used to estimate binding ability through the binding enthalpy (ΔH) [34]. An ITC isotherm of the CE1/BmPSI system was performed at 25 °C with an cell volume of 1.4 mL using a stirring rate of 307 rpm. The CE1 probe was injected 2 μL at a time into a solution of BmPSI at 210 s to allow complete equilibration of the CE1/BmPSI system between each injection. The most appropriate experimental conditions were grouped with the experiment between wild-type BmPSI and the CE1 probe. Then, the other amino acid mutants with the same concentration were experimented on under the same conditions. The differences in binding ability among these four proteins were determined from the curves constructed during these ITC experiments.

## Figures and Tables

**Table 1:** The energetic profile of the interaction of the BmPSI with CE1 obtained from ITC.

Molecule	ΔH (cal/mol)	ΔS (cal/mol/deg)	*n*	K (M^−1^)
BmPSI	−3.3 × 10^4^ ± 269.2	−77.9	1.02	1.74 × 10^7^

**Table 2:** Primer sequences of modifications on *Bmpsi*.

Name	Primers
Bmpsi	5′-cgcggatccatgagtgattattcttctat-3′,
	5′-ccgctcgagtcactgctggtggtcggagc-3′
ΔKH_1-1	5′-cgatcagagcatcaatattgtgaatcatcgaggaa-3′
	5′-ttcctcgatgattcacaatattgatgctctgatcg-3′
ΔKH_1-2	5′-ccaggccctaatgcaatgctcctcgccaacaagga-3′
	5′-tccttgttggcgaggagcattgcattagggcctgg-3′
ΔKH_1-3	5′-aacggactcgccaccactcttatatctagtgtcaa-3′
	5′-ttgacactagatataagagtggtggcgagtccgtt-3′
ΔKH_1-4	5′-gaccgaccagagatgcgcaaagttggtgggcctgt-3′
	5′-acaggcccaccaactttgcgcatctctggtcggtc-3′
I116G	5′-gttggactaggaattggacgtggt-3′
	5′-accacgtccaattcctagtccaac-3′
L127G	5′-atcaccagagggcaagcagaatcc-3′
	5′-ggattctgcttgccctctggtgat-3′
K134G,	5′-tccggttgcgggatacaaatggca-3′
	5′-tgccatttgtatcccgcaaccgga-3′
A161G	5′-atacagagaggtaaagaattagtg-3′
	5′-cactaattctttacctctctgtat-3′
L164G	5′-gctaaagaaggagtgaaccaaatt-3′
	5′-aatttggttcactccttctttagc-3′
V165G	5′-aaagaattagggaaccaaattgtg-3′
	5′-cacaatttggttccctaattcttt-3′
IGGI 117, 118, 121, 124 AAAA	5′-ggactaatagctgcacgtggtgga-3′
	5′-tccaccacgtgcagctattagtcc-3′,
	5′-cacgtggtgcagaacaagccaccagactgca-3′
	5′-tgcagtctggtggcttgttctgcaccacgtg-3′,
	5′- gcagaacaagccaccagactgcaa-3′
	5′-ttgcagtctggtggcttgttctgc-3′

**Table 3:** Primer sequences of modifications on *Slpsi*

Name	Primers
*Slpsi*	5′- cgggatccatgagtgattattcttctatggc-3′
	5′-ggaattcctattgatgatggtcggg-3′
I116G	5′-atggttggactcggaattgggcgcggc-3′
	5′-gccgcgcccaattccgagtccaaccat-3′
L127G	5′-atcacacgcgggcaggcggagtca-3′
	5′-tgactccgcctgcccgcgtgtgat-3′
IGGI 117, 118, 121, 124 AAAA	5′-ggactcatagctgggcgcggcggtgag-3′
	5′-ctcaccgccgcgcccagctatgagtcc-3′,
	5′-ggactcatagctgcacgcggcggtgag-3′
	5′-ctcaccgccgcgtgcagctatgagtcc-3′,
	5′-gcacgcggcgcagagcaggccacacgc-3′
	5′-gcgtgtggcctgctctgcgccgcgtgc-3′

## Abbreviations

dsx	Doublesex
PSI	P-element somatic inhibitor
CD	Circular dichroism
ITC	Isothermal titration calorimetry
EMSA	Electrophoretic mobility shift assay
